# Risk perception before and after presymptomatic genetic testing for Huntington's disease: Not always what one might expect

**DOI:** 10.1002/mgg3.494

**Published:** 2018-11-04

**Authors:** Kelsey Stuttgen, Rachel Dvoskin, Juli Bollinger, Allison McCague, Barnett Shpritz, Jason Brandt, Debra Mathews

**Affiliations:** ^1^ Berman Institute of Bioethics Johns Hopkins University Baltimore Maryland; ^2^ Institute of Genetic Medicine Johns Hopkins University School of Medicine Baltimore Maryland; ^3^ Department of Psychiatry and Behavioral Science Johns Hopkins University School of Medicine Baltimore Maryland; ^4^ Department of Neurology Johns Hopkins University School of Medicine Baltimore Maryland

**Keywords:** genetic counseling, genetic testing, Huntington’s disease, risk perception

## Abstract

**Background:**

In 1983, Huntington's disease (HD) was the first genetic disease mapped using DNA polymorphisms. Shortly thereafter, presymptomatic genetic testing for HD began in the context of two research studies. One of these trials was at the Johns Hopkins University Huntington's Disease Center.

**Methods:**

As part of the protocol, risk perception (RP) values were collected at 16 time points before and after testing. The current study investigated changes in RP scores before and after genetic testing. Of the 186 participants with pre‐ and post‐testing RP values, 39 also had contemporaneous research clinic notes and recent semi‐structured interviews available for analysis.

**Results:**

The data reveal tremendous diversity in RP. While the RP scores of most individuals change in the way one would expect, 27% of participants demonstrated unexpected changes in RP after disclosure. A significantly higher proportion of individuals who received an expanded repeat result had unexpected changes in RP, compared with those who received normal repeat results.

**Conclusions:**

The data suggest that individuals’ RP is influenced by more than merely the results of genetic testing. This finding is important for genetic counselors and healthcare providers, as it suggests that even comprehensive patient education and disclosure of genetic test results may not ensure that people fully appreciate their disease risk.

## 1 INTRODUCTION

Huntington's disease (HD) is an autosomal dominant inherited condition that is caused by a trinucleotide repeat (CAG) expansion on chromosome 4 (at locus 4p16.3). This progressive neurodegenerative condition is characterized by cognitive deterioration, involuntary movements, abnormal voluntary motor control, and affective symptoms. While some symptoms of the disease can be managed with medication, there is no cure.

Genetic testing via linkage analysis became available for HD in 1986, when the Johns Hopkins University (JHU) and Massachusetts General Hospital began research studies to provide presymptomatic testing for those at risk. The JHU trial continued through 1998 but after the isolation of the gene responsible for HD in 1993 (MacDonald, 1993), linkage analysis was replaced by direct mutation analysis (direct testing). Compared to linkage analysis, which was 95%–99% accurate, direct testing is certain. Since HD is a fully penetrant condition when more than 39 CAG repeats are present, test results in that range guarantee that individuals will develop the disease should they live until the age of onset. Conversely, test results of a normal repeat mean that one definitely will not develop the disease.

How people perceive their risk and their responses to it play an important role in individuals’ decision‐making processes and psychological well‐being. Prior studies have investigated changes in risk perception (RP) before and after presymptomatic testing for hereditary cancer and Alzheimer's disease (Aspinwall, Taber, Kohlmann, Leaf, & Leachman, 2013; Butow, Lobb, Meiser, Barratt, & Tucker, 2003; Gurmankin, Domchek, Stopfer, Fels, & Armstrong, 2005; Schüz, Schüz, & Eid, 2013), but, unlike HD, these are not fully penetrant conditions. Additionally, in the case of hereditary cancer, there are steps that can be taken to decrease the chances of developing cancer, including enhanced screening, prophylactic surgery, and chemoprevention (McLaughlin et al., 2007). No steps can be taken to decrease the risk of developing HD. While several studies have explored the effects of presymptomatic HD testing (Brandt, Quaid, & Folstein, 1989; Crozier, Robertson, & Dale, 2014; Meiser & Dunn, 2000), to our knowledge only three studies (Binedell, Soldan, & Harper, 1998; Codori & Brandt, 1994; Decruyenaere et al., 1999) measured individual's perceived risk for HD before testing and after testing. Findings by Codori and Brandt (1994) showed significant differences between the mean disclosed risk and the mean perceived risk among individuals who received results of an expanded repeat but not among individuals who received results of a normal repeat. Findings by Binedell et al. (1998) showed that at‐risk individuals who pursue presymptomatic testing perceive themselves as more likely to carry an expanded repeat than individuals who do not pursue presymptomatic testing. Additionally, findings by Decruyenaere et al. (1999) showed that higher pre‐test perceived risk for HD is positively correlated with depression.

While never published, individuals’ RP scores were solicited and recorded at each visit—up to eleven years after testing—from individuals in the JHU HD presymptomatic protocol. The current study analyzed long‐term changes in RP, conducted semi‐structured follow‐up interviews, and reviewed contemporaneous research clinical notes from individuals who participated in the JHU HD presymptomatic protocol, to investigate factors that contributed to changes in RP. To our knowledge, this is the first study to measure long‐term changes in RP for a genetic disease.

## 2 METHODS

### 2.1 Editorial policies and ethical considerations

This study was approved by and conducted in accordance with the policies and procedures of the Johns Hopkins IRB. Written informed consent was obtained from all participants included in the study (both at the time of presymptomatic testing and again for recent interviews).

### 2.2 Data collection

**Figure 1:**
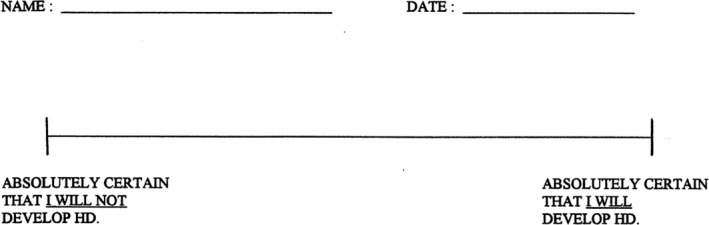
Visual analog scale. Participant indicated his/her perceived risk on the horizontal line. The line spans 100 mm, and a risk perception percentage was calculated by measuring the distance in millimeters of the marking indicated by the participant from the leftmost side of the line

Risk perception was measured using a 100 mm visual analog scale (Figure 1). The left anchor was labeled “absolutely certain that I will not develop HD,” while the rightmost was labeled “absolutely certain that I will develop HD.” Participants indicated their perceived risk by putting a vertical slash along the horizontal line. RP was calculated as number of millimeters from the left anchor.

Risk perception was collected at baseline, which was the participant's first research appointment and prior to genetic testing. RP was also assessed at 3 months, 6 months, 9 months, 12 months, 18 months, 24 months, 30 months, 3 years, and then annually, up to 11 years post‐disclosure. The average number of data points available per participant included in the analysis was 7.1, where the minimum number of data points required for inclusion was two—a baseline RP score plus at least one RP score after genetic testing.

### 2.3 Participants

Risk perception data were collected on 214 participants who were enrolled in the JHU HD presymptomatic protocol from 1986 to 1998. Of these 214 participants, 28 were eliminated from analysis due to an insufficient number of data points. Sample characteristics of the 186 individuals included in the analysis are shown in Table 1. Sample characteristics of the subset of 39 individuals who had contemporaneous research clinic notes and recent semi‐structured interviews are shown in Table 2.

**Table 1:** Sample characteristics of 186 individuals included in risk perception analysis

	Male	Female	Expanded repeat	Normal repeat	Other	Total
*N* (%)	78 (41.9)	108 (58.1)	56 (30.1)	115 (61.8)	15^a^ (08.1)	186

a Consists of individuals who were uninformative by linkage testing and individuals who were undisclosed, meaning they did not receive their genetic test results, but participated in pre‐test counseling and appointments over many years at the same time intervals as those who received genetic test results.

**Table 2:** Sample characteristics of individuals in follow‐up study

	Male	Female	Expanded repeat	Normal repeat	Other	Total
*N* (%)	19 (48.7)	20 (51.3)	15 (38.4)	21 (53.8)	3^a^ (07.7)	39

a Consists of individuals who were uninformative by linkage testing and individuals who were undisclosed, meaning they did not receive, their genetic test results but participated in pre‐test counseling and appointments over many years at the same time intervals as those who received genetic test results.

### 2.4 Data analysis

Changes in RP were placed in two categories: expected changes and unexpected changes. Expected changes were defined as a decrease in RP after a normal repeat result, an increase in RP after an expanded repeat result, or no change in RP after an uninformative result or no result. Unexpected changes were defined as increased RP after receiving a normal repeat result, decreased RP after receiving an expanded repeat result, no change in RP after receiving a test result, variation in RP after receiving a test result, and change in RP after receiving an uninformative result or no result.

As part of a follow‐up study investigating long‐term effects of presymptomatic genetic testing, a subset of individuals who were enrolled in the JHU HD presymptomatic testing protocol were interviewed once between 2015 and 2017, which was 20–30 years after presymptomatic testing was performed. These semi‐structured interviews lasted approximately one hour, and topics included individuals’ testing experience, reactions to test result, confidence in results, and impact of testing on mental health, relationships, and life decisions. The interviews were audio recorded, transcribed, and scrubbed of all identifying information. Research charts of all participants who were interviewed in the follow‐up study were obtained and analyzed. A codebook was developed and included codes for RP, confidence in result, change in RP after testing, and risk misperception. All interviews and charts were double coded, and any conflicts in coding were discussed by the coders and reconciled. QSR International's qualitative analysis Software NVivo 11 was used for data analysis, along with manual analysis of data.

Code reports generated from the research charts, research records, and interviews of the 39 individuals who participated in the follow‐up study were analyzed for discussion about RP. Coded RP content was classified into one of 13 categories (Table 3).

**Table 3:** Factors that appear to influence risk perception

Factors	Description	Representative quote	Number of individuals who expressed factor
Symptomatizing	Worry that normal failures (e.g., dropping keys) are symptoms of HD	“I think everybody who is related to somebody that has it, thinks of every time they move their foot, or do a tick, or hesitate with what they're saying, they feel like they have it. At least all the conversations I've had with people. It's just sort of natural. So, I was almost certain that I had it.”	9
Unable to accept normal repeat result	Inability to accept that one has a normal repeat and is no longer at risk for HD	“And it wasn't that I didn't trust the test result because I really did trust the accuracy and, you know, the things that Hopkins had in place, you know, that it was an accurate test result. It wasn't that. I just couldn't believe that that's what it was.”	5
Genetic test results of family members	Test results of family members may be an indication of one's own test results (e.g., Individual believes it is more likely s/he has an expanded repeat after learning his/her family member has a normal repeat)	“My brother‐in‐law was very much on the, 'No, she doesn't have it.' And I was on the over 50 percent, like, 'Yeah, I do.' My sister had been tested, she didn't have it. What are the chances of both of us not having it? You know?”	4
Hope for a cure	Hope for a cure to be developed before one develops symptoms of HD	“There is hope for a cure.”^a^	3
Linkage test accuracy	Lower accuracy of linkage test (96%–99%) compared to direct test (100%)	“After they had identified the gene, they asked to retest me [with the direct test] which they did, and I didn't ask for the results. Every year when I would come down they would give you the same sort of questionnaires. On one of the questionnaires was just a line on a piece of paper that someone says I have Huntington's disease and this one says I don't have Huntington's disease, and you had to put an x. I could never put my x… it was just beside I don't think I have it. I could never put it on ‘I don't have it.’ That's the only thing I couldn't do. I got close to there but not – I couldn't. I never asked for the [direct test] results.”	3
Physical resemblance to affected family member	Physical resemblance to an affected family member increases one's likelihood of having an expanded repeat	“I thought, ‘Oh my gosh, if I look my mother, then I must have her gene for Huntington's.’”	3
Optimism after expanded repeat result	Hope that one will not develop HD after receiving results that one carries an expanded repeat	Interviewer: “Can I ask you when they gave you your results, did you think you would get it? Did you believe the results? Or were you optimistic that you wouldn't?” Participant: “I was optimistic I wouldn't.”	3
Personality resemblance to affected family member	Personality resemblance (e.g., a bad temper) to an affected family member increases one's likelihood of having an expanded repeat	“I always felt like I was like my mother in her values, in her way she was with people. My mother, if you did something to her she held a grudge forever. I felt like I was like her, so maybe I did have some of her.”	2
Belief in lab mistake	Belief one's test result is incorrect due to a laboratory mistake	"Yeah, when I first understood I didn't carry the gene, there's still always that apprehension, 'Well, what if the lab was wrong?'"	2
Misunderstanding genetic test results	Confusion regarding whether “positive” result meant good news or gene‐positive (expanded repeat)	“They said, 'you tested positive.' And I literally didn't know whether that meant that I had the gene or positive like it's a positive result or a positive outcome.”	1
Misunderstanding HD Risk	Misunderstanding each child born to a parent affected by HD has a 50% chance of having an expanded repeat, or misunderstanding how HD is passed on in families	“There's some disease that runs in the family, that turns your brain to mush but don't worry about it because it skips a generation usually.”	1
Age of parental onset	Increased worry of developing HD as one approaches the age of onset of his/her affected parent	“He has been increasingly worried about developing HD because he is now the age his mother was when she began to show symptoms.”	1
Denial after expanded repeat result	Inability to accept that one will develop HD after receiving results that one carries an expanded repeat	“How could those tests be positive, there must have been a mistake, those things happen every day, right?”	1

Note

HD: Huntington's disease; RP: risk perception.

Factors are defined and represented with a quote taken either from clinic notes or interviews.

a Quote was taken from visual analog scale; individual with expanded repeat and lower than expected RP wrote this next to her indicated RP.

## 3 RESULTS

**Figure 2:**
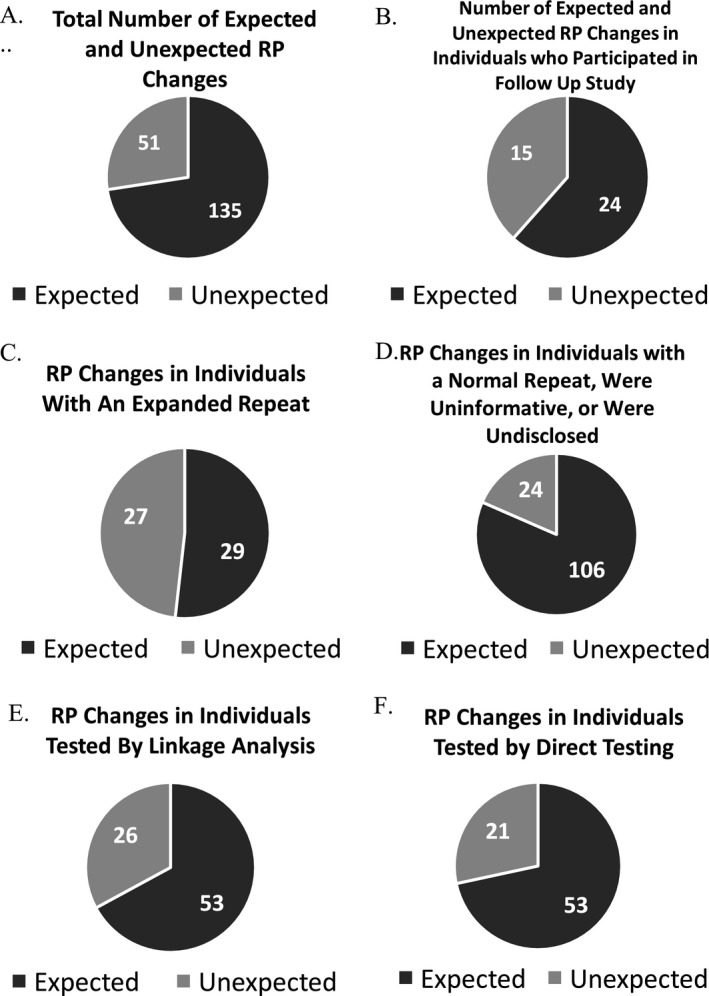
(a) Total number of expected and unexpected risk perception (RP) changes. (b) Number of expected and unexpected changes in individuals who participated in the follow‐up study. (c) RP changes in individuals with an expanded repeat. (d) RP changes in individuals with a normal repeat were uninformative, or were undisclosed. (e) RP changes in individuals tested by linkage analysis. (f) RP changes in individuals tested by direct testing

Fifty‐one of 186 (27%) participant's RP scores demonstrated unexpected changes (Figure 2a). No significant differences in unexpected changes were observed between females and males. However, a significantly higher proportion of individuals who received an expanded repeat result (27/56; 48%) had unexpected changes, compared with those who received a normal repeat result, uninformative result, or were undisclosed (24/130; 20%) (*x* ^2^ = 17.4, *p* = 0.00003) (Figure 2c,d). Unexpected changes were also observed at a higher rate in individuals tested by linkage (26/79; 33%) than in individuals tested by direct testing (21/74; 21%), but these differences were not significant (Figure 2e,f).

Factors that appear to influence RP identified in interviews and clinic notes are listed and described in Table 3. Of the participants in the long‐term follow‐up study (*n* = 39), 27 participants identified one of more of these factors (12 participants did not discuss RP).

## 4 DISCUSSION

Unexpected changes in RP were observed in more than 1/4 of the full cohort, indicating perceived risk is influenced by more than genetic test results alone. This finding is concordant with other studies, which have found that RP is complex and influenced by a number of factors that may impair accurate risk comprehension (Croyle & Lerman, 1999; Hopwood, 2000). In a literature review by Sivell et al. (2007), 59 studies presenting data on the way individuals perceive, construct, and interpret risk for a range of diseases were evaluated. Nineteen of the studies investigated how individuals construct RP by considering the factors and beliefs on which people base their RP. These factors included past experiences (d'Agincourt‐Canning, 2005; Hallowell, Statham, & Murton, 1998; Kelly et al., 2004; Kenen, Arden‐Jones, & Eeles, 2004; Robertson, 2000), environmental factors (Gorin, & Albert, 2003; Ryan & Skinner, 1999), occupation as it relates to carcinogen exposure (Liede et al., 2000), diet (Ryan & Skinner, 1999; Wilson et al., 2005), stress and worry (Ryan & Skinner, 1999), physical resemblance to affected relative (Fanos & Gatti, 1999), and genetic and family history factors (Gorin & Albert, 2003; Julian‐Reynier et al., 1998; Liede et al., 2000; Ryan & Skinner, 1999; Wilson et al., 2005). Decruyenaere et al. (1999) also found that parental age of onset of HD informed an individual's RP.

**Figure 3:**
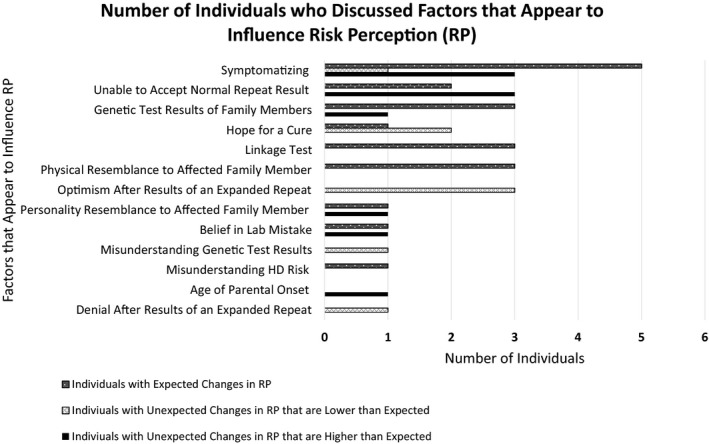
Factors that appear to influence risk perception and the number of individuals who discussed each factor

Analysis of additional data from a subset of the full cohort, including both contemporaneous clinic notes and recent interviews identified both previously published and novel factors that appeared to affect RP. Novel factors included symptomatizing, personality resemblance to affected family member, inability to accept normal repeat result, misunderstanding genetic test results, misunderstanding HD risk, genetic test results of family members, optimism after results of an expanded repeat, denial after results of an expanded repeat, linkage testing, belief in lab mistake, and hope for a cure. As shown in Figure 3, these factors were discussed by both those with unexpected and those with expected changes in RP. Of note, we are defining “factors that appear to influenced RP” as those factors that seem to have influenced how individuals reported their risk on the visual analog scale (Figure 1). Individual's self‐reported disease risk may be influenced not only by their understanding of their genetic test results, but also by other factors, such as coping mechanisms and anxiety. For example, two participants who received an expanded repeat result demonstrated a lower than expected RP score stated they were hopeful a cure would become available and they would not develop symptoms of HD (Figure 3). Further, three participants who received a normal repeat result demonstrated higher than expected changes in RP stated they worried normal human failures (e.g., dropping keys) were symptoms of HD (Figure 3).

Data from the current study indicate that people often appear to misunderstand their risk. A higher proportion of unexpected changes in those with expanded repeats may indicate denial or difficulty accepting a disease gene‐positive result, or rather may be an expression of hope about their prospects in the face of the expanded repeat finding. The unexpected RP scores observed in this population may be a manifestation of coping mechanisms being employed after receiving a very difficult test result. Prior studies have shown that minimization of risk and/or denial is common processing strategies after receiving health risk information (Aspinwall et al., 2013; Mathews, Fins, & Racine, 2017; Meiser & Dunn, 2000). Additionally, if the receipt of negative information puts a person in a negative mood, they are less likely to process a health message associated with a disease and their potential of having the disease (Agarwal & Teas, 2001). Finally, interviews with participants in phase I or II oncology trials revealed that many individuals believe having positive thoughts or expressions will improves one's chances of personally benefitting from a therapy or cure (Sulmasy et al., 2010). This belief may be shared by individuals who carry expanded repeats for HD.

Individuals undergoing presymptomatic testing for HD typically undergo two pre‐test genetic counseling sessions before receiving test results. Individuals in the JHU HD presymptomatic study received pre‐test counseling, but since they were the first cohort to be tested presymptomatically, five pre‐test appointments were required before receiving test results. The proportion of unexpected changes observed in a cohort well‐counseled and well‐educated on both HD and genetic testing for HD is surprising.

## 5 CONCLUSIONS

After receiving results of genetic testing, people's RPs do not always change in the way one would expect. Data from this study and others indicate that an individual's RP is complex and influenced by a variety of factors which are likely not only disease‐specific but also specific to an individual's past experiences and beliefs. Furthermore, RP may play an important role in an individual's decision‐making processes and psychological well‐being.

This study suggests that extra steps may be necessary to ensure individuals are processing and adapting well to their test results. Without disabusing people of reasonable hope in the face of difficult news, possible interventions include post‐test counseling appointments with discussions about RP and continued education about HD. Further, research is needed to investigate factors that influence perceived risk. Findings from this study should be considered by both genetic counselors and healthcare providers, and efforts should be made to respond to RP in order to improve patients’ experience of testing and overall well‐being.

### 5.1 Study limitations

The study population included self‐selected individuals who were not representative of all individuals eligible for testing. Claes, Denayer, Evers‐Kiebooms, Boogaerts, and Legius (2004) suggested that individuals who present for genetic testing may have a higher perceived ability to understand and cope with genetic test results. Not all participants in the current study were independent, meaning some participants in the study were blood relatives of other participants in the study; this could lead to family‐specific effects. Also, individuals in this study were primarily Caucasian; further research is needed to assess possible differences in RP across different cultural backgrounds and ethnicities.

Since the factors we identified were not exclusively discussed by individuals with unexpected results, we cannot conclude these factors are solely responsible for the observed unexpected changes in RP. It is also important to note that almost all prior studies that have investigated factors that inform RP have been on hereditary cancer. Therefore, the novel factors identified in this study could be due to differences between hereditary cancer and HD.

As part of the JHU HD presymptomatic protocol, individuals received extensive pre‐ and post‐test counseling. Thus, results of this study may not be comparable to individuals who receive the current standard of two counseling sessions prior to presymptomatic HD genetic testing. Additionally, individuals in this study underwent genetic testing between 1986 and 1998. These were some of the first individuals to undergo genetic testing, and trust in genetic test results and understanding of genetic information may be different now than it was at that time. Finally, this study focused on RP in the context of HD, so caution should be used when generalizing these findings to other conditions.

## CONFLICT OF INTEREST

All of the authors declare that they have no conflict of interests.
